# Implementing ultrasound in emergency medical services: assessing physician proficiency and training requirements

**DOI:** 10.1186/s13049-025-01391-6

**Published:** 2025-05-20

**Authors:** C. Engelen, J. Haack, D. Lämmermann, W. Hitzl, J. C. Kubitz, G. Breuer, A. Kamphausen, T. Hübner

**Affiliations:** 1 Department of Anesthesia and Intensive Care Medicine, Paracelsus Medical University, Nuremberg, Bavaria Germany; 2 Emergency Departement, Lauf/Pegnitz Hospital, Lauf/Pegnitz, Bavaria, Germany; 3 Department of Anesthesia, Sana Kliniken Oberfranken, Coburg, Bavaria Germany; 4 Research and Innovation Management (RIM), Team Biostatistics and Publication of Clinical Trial Study, Paracelsus Private Medical University, Salzburg, Austria; 5 Department of Emergency Medicine, ANregiomed, Ansbach, Bavaria Germany

**Keywords:** Emergency medical services, POCUS, Sonography, Prehospital, Training

## Abstract

**Background:**

Bedside ultrasound plays an important role in diagnostics and monitoring, especially in emergency medicine. Modern technology makes ultrasound available in a mobile and portable form, so it can be used even in prehospital emergency care with several interventional and diagnostic applications. This also raises the question of what kind of education and training is necessary for EMS (emergency medical services) physicians to be able to use Point-of-Care Ultrasound (POCUS) in the prehospital setting.

**Aims:**

This observational study investigates the use of prehospital POCUS in a rural EMS area. It focuses the question of what level of competence is needed for EMS physicians to use POCUS adequately in the prehospital emergency setting for correct application and interpretation of the findings.

**Method:**

This was a quality assurance measure designed as a prospective cohort study. We investigated POCUS examinations performed by EMS physicians in the EMS Service Area of Nuremberg City, Germany between June 2021 and July 2022. Patients transported to three specific hospitals in Nuremberg city after care were followed up and the prehospital findings were compared with the in-hospital radiological results. The number of correct findings was correlated with the level of competence in POCUS examinations of the performing EMS physicians. Various classifications of competence were used to assess the influence of training and education on the safe application of prehospital POCUS.

**Results:**

Two hundred fifty-eight prehospital POCUS examinations were documented, with 108 followed up, including 268 sonographic findings. There was a wide range of indications for POCUS use. In 79.5% of cases the prehospital findings correspond with those in-hospital. By correlating the correct findings with the participants level of competence, there was no significant difference between POCUS-experienced and -inexperienced EMS physicians, even when divided into different categories.

**Conclusion:**

POCUS can be used in prehospital emergency care for a wide range of indications safely, with a high number of correct diagnoses and findings. Our results suggest that emergency POCUS is easy to learn and EMS physicians do not need intensive training to perform POCUS adequately in the prehospital setting.

**Take home messages:**

- Mobile ultrasound appears to be useful in the prehospital setting
- It can be used by EMS physicians even without extensive prior experience and expertise
- POCUS is able to find important findings for prehospital patient’s care with a high level of certainty

## Introduction

Bedside ultrasound is an integral part of modern patient care and plays an important role in diagnosing and monitoring various diseases and traumatic injuries. In emergency medicine in particular it is essential to respond quickly to identify the patient’s acute problem for optimal care. Using bedside targeted ultrasound examinations (POCUS – Point-of-Care Ultrasound) is a crucial component of clinical diagnostics.

The continuous development of technology of mobile ultrasound devices enables their use in prehospital care as well. More than 10 years ago, prehospital POCUS was cited as one of the top five research topics in emergency medicine [1]. Since then, ultrasound diagnostic in prehospital emergency medicine has been increasingly mentioned, discussed and a variety of research on different aspects of its use has been published. A large number of publications addressed the implementation and use of ultrasound in the prehospital setting, not only for diagnostic purposes but also for assisting interventional procedures, such as vascular access [2–7]. One particular focus of interest is the need for education and training [8–10]. Across this research, participants typically received intensive training in prehospital POCUS examinations, either at the beginning or repeatedly during data collection. The question of what is necessary to gain competence in POCUS examination has been analyzed, and different concepts and training courses have been published. One of these, named pPOCUS (prehospital Point-of-Care Ultrasound), is currently published as a training concept for EMS physicians in Germany [11].

Despite a series of reported experiences, the organization and intensity of training required to enable the use of emergency ultrasound in patient care remains an open question. Some publications name a certain number (e.g. 50–100) of examinations to be performed during training [12]. However, in addition to the question of acquiring skills, there is also the question of how to maintain these skills over time. A large part of EMS physicians in Germany do not necessarily perform emergency ultrasound examinations regularly during their work in-hospital. The mere availability of mobile ultrasound in the prehospital setting does not necessarily ensure that sufficient conclusions can be obtained from the investigation.

As the use of POCUS is not yet part of EMS physicians medical training programs and considering the wide spectrum of physicians working in emergency medicine in Germany, it seems important to know how POCUS competence can be achieved and sustained over time, especially regarding the fact that mobile ultrasound devices are rolled out extensively.

This prospective observational study investigated the implementation of POCUS examinations in prehospital emergency care with regard to the question about what expertise and training EMS physicians need for appropriate utilization.

## Methods

This study was a quality assurance measure designed as an observational prospective clinical trial. It adhered to the principles outlined in the Declaration of Helsinki, and its conduct was reviewed and approved by the institutional review board of PMU – Paracelsus Medical University, Nuremberg, Germany (IRB-2021–036).

**Fig. 1:**
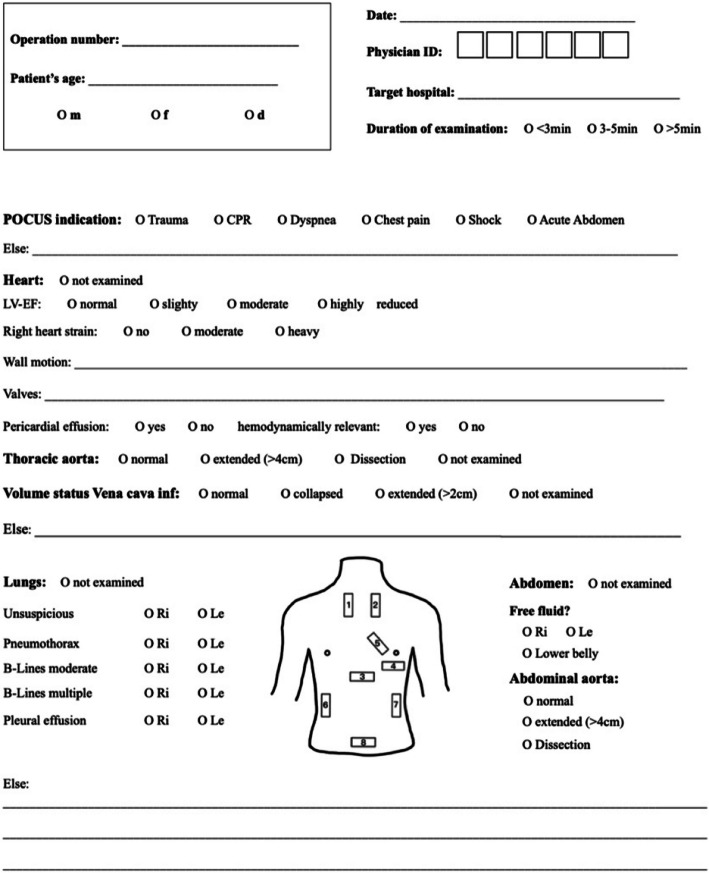
Documentation form used for documenting POCUS findings

In May 2021, the four EMS physician ambulance cars of the Nuremberg City Ambulance Services were equipped with mobile ultrasound devices (GE Vscan Extend, GE Healthcare). Between June 2021 and July 2022, prehospital POCUS examinations performed by EMS physicians were recorded on a documentation form (Fig. 1) for prospective exploratory data collection. All patients who underwent ultrasound examination and were transported to one of the three sites of Nuremberg City Hospital—Campus South (Level III care), Campus North (Level II care) and Hospital Lauf/Pegnitz (Level I care)—for further treatment were followed up regarding the radiological and sonographic findings obtained there. If a patient was left on site after emergency care or transported to another Nuremberg hospital, data were not available and not followed up.

### Ultrasound diagnostics in prehospital emergency care

The indication for performing POCUS on site was left to the attending EMS physician. There was no randomization or blinding. In addition, we asked the indication for POCUS use on scene and the time spent on the patient’s investigation (less than 3 min, 3–5 min or more than 5 min).

Table 1 shows the overview of type and characteristics of prehospital POCUS findings on the documentation form, which was created by the research group and displayed on each rescue station on paper (Fig. 1).

**Table 1:**
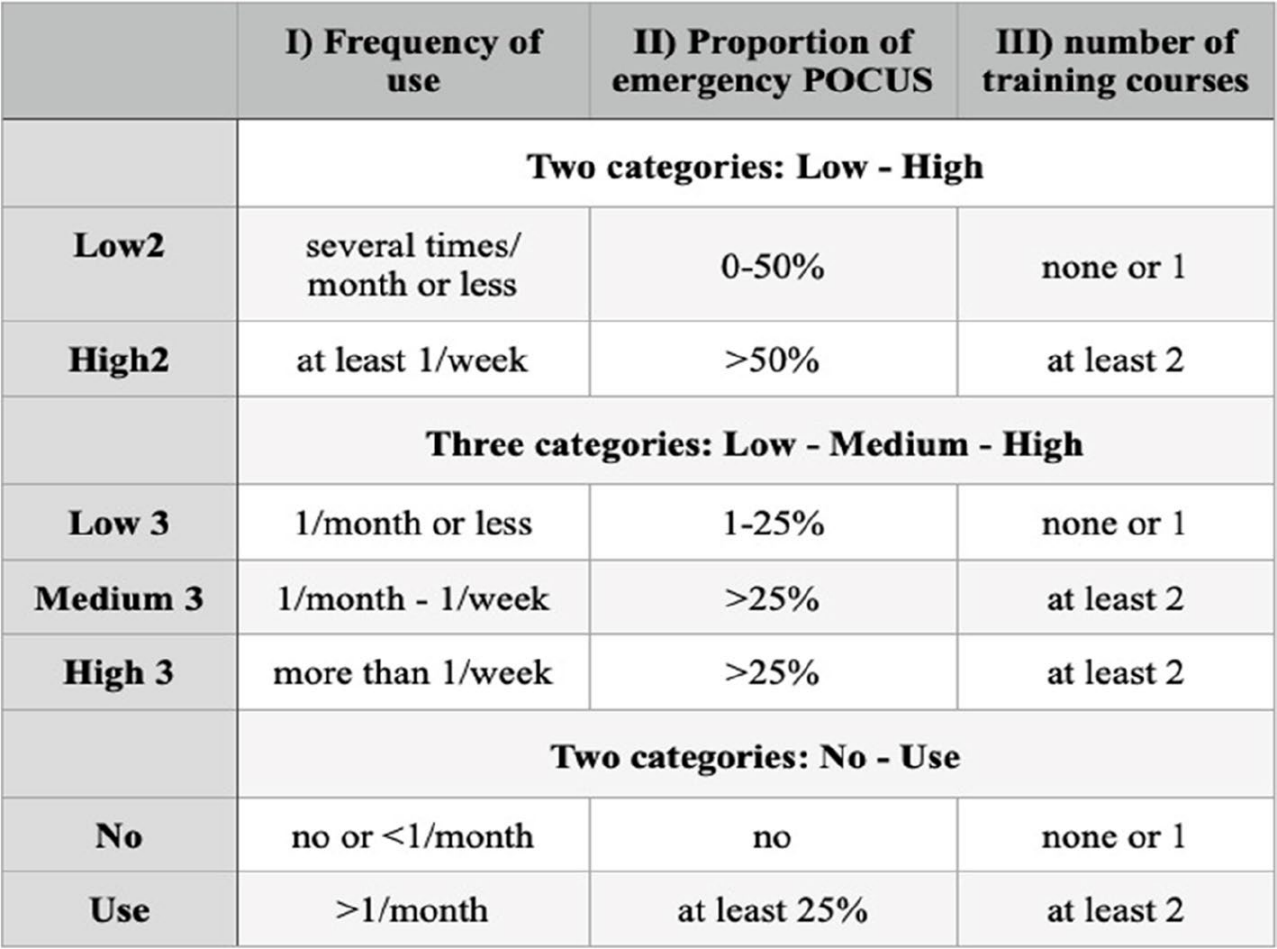
The three different categorizations of competence in emergency POCUS examinations

### POCUS competence of participating EMS physicians

To compare the different levels of competence in emergency POCUS examinations, a scoring system was developed to categorize the participating emergency physicians. This scoring was based on the consensus of five independent experts in emergency medicine. None was involved in data collection or implementation of this study. The following aspects were included:

I) *Frequency of use of ultrasound in daily clinical practice:*
  Less than 1/month - 1/month - several times/month - 1/week - several times/week - daily use - several times/day
II) *Proportion of emergency POCUS examinations (estimated)*
  0% - 1–25% - 26–50% - 51–75% - 76–100%
III) *Number of attended POCUS courses and training concepts *(such as FAST, FATE, FEEL, RUSH…) 0 - 1/2 - >2

Based on this categorization, a total of three different classifications of participants were made according to their ultrasound competence (Table 2).

**Table 2:**
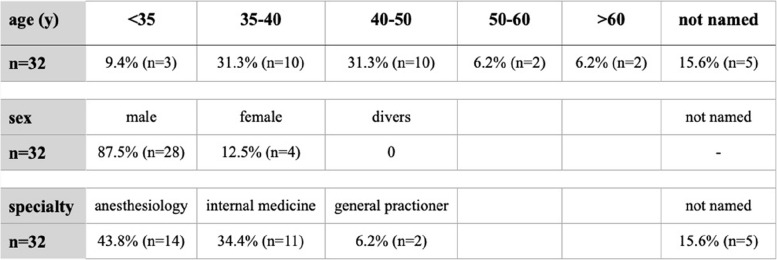
Epidemiological data of EMS physicians that took followed up examinations

All prehospital ultrasound findings were compared with regard to the radiological findings obtained in-hospital (ultrasound examination, X-ray or CT scan during emergency room treatment). It was documented whether the in-hospital findings corresponded with those from prehospital assessments and whether these were therefore correctly recorded during the prehospital POCUS examination.

### Statistical methods

Data was checked for consistency. Fisher’s Exact test or Pearson’s Chi-Squared test were used to analyze cross tabulations. All reported tests were two-sided, and *p*-values < 0.05 were considered statistically significant. All statistical analyses in this report were performed by use of STATISTICA 13 (Hill, T. & Lewicki, P. Statistics: Methods and Applications. StatSoft, Tulsa, OK).

## Results

### Cohort

A total of 32 EMS physicians participated in data collection. The physician’s epidemiological data is shown in Table 2.

A total number of 258 prehospital POCUS examinations were documented during the observation period. In 108 (41.5%) of these, the in-hospital findings were available and followed up. All other documented patients (*n* = 150) were either left at home after treatment or admitted to a hospital where in-hospital data was not available.

The median age of the examined and followed up patients (*n* = 108) was 66.4 years (1;90), 44.9% were female (*n* = 59).

### Ultrasound diagnostics in prehospital emergency care

A total number of 268 POCUS findings were recorded during the 108 sonographic examinations. The comparison with in-hospital radiological examinations showed a high degree of correct findings in the performed prehospital ultrasound. In 79.5% of the POCUS examinations (*n* = 210), the documented prehospital findings corresponded to those in-hospital.

In 65.7% (*n* = 71) of all cases, less than 3 min were required to perform the POCUS examination, in 28.7% (*n* = 31) it took 3–5 min, none was more than 5 min, 5.6% (*n* = 6) of cases time was not documented.

**Fig. 2:**
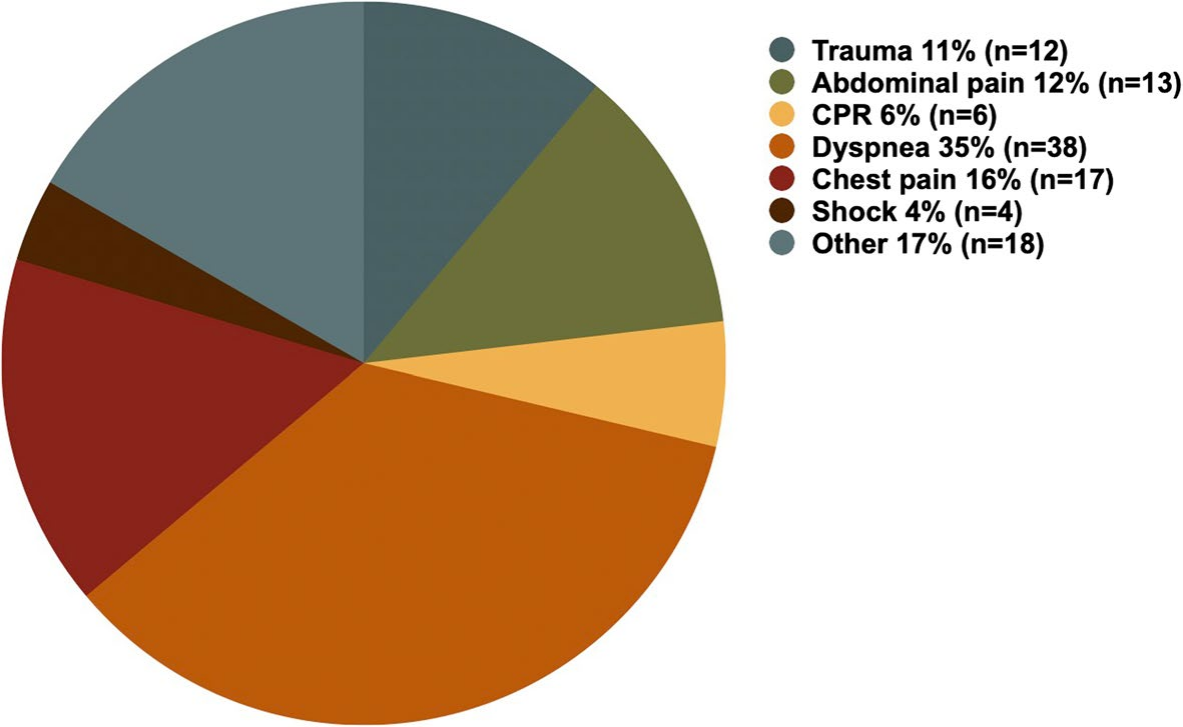
Indications for use of prehospital ultrasound diagnostics (other n=18: iv access n=6, not named n=12)

The distribution of indications for POCUS use is shown in Fig. 2 (*n* = 108).

A listing of all prehospital POCUS findings and the number of identical results in in-hospital diagnostics is shown in Table 3.

**Table 3:**
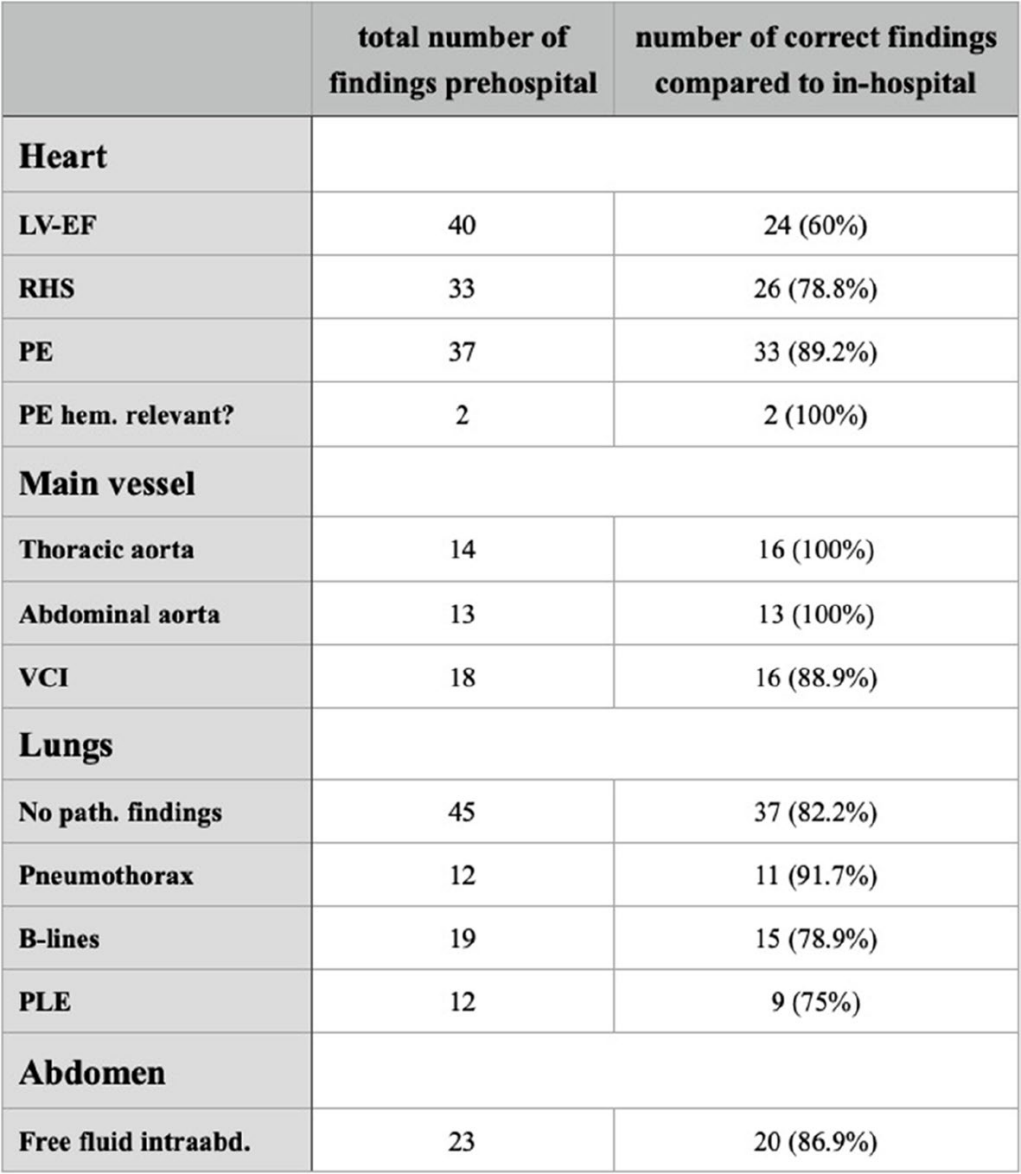
Overview of followed-up POCUS findings and correct prehospital findings

*LV-EF* Left-ventricular ejection fraction, *RHS* Right heart strain, *PE* Pericardial effusion, *hem.* Hemodynamically, *VCI* Vena cava inferior, *path.* Pathological, *PLE* Pleural effusion, *intraabd.* Intra-abdominal

### POCUS competence of participating EMS physicians

Data about attended training courses and the usage of ultrasound was available from 28 EMS physicians. As shown in Table 1 scoring system, the participants were rated according to their level of competence in the use of ultrasound.

When divided into two levels of competence (named High2 and Low2), we had *n* = 7 (25%) in Low2 and *n* = 21 (75%) in High2. By dividing into three levels of competence (Low3, Medium3 and High3), we found *n* = 4 (14%) in Low3, *n* = 12 (43%) in Medium3 and *n* = 12 (43%) in High3.

By dividing into two categories (named *no use* and *use*), we found *n* = 2 (7%) in *no use* group and *n* = 26 (93%) in *use* categorized.

### Number of correct POCUS findings compared with level of competence

**Fig. 3:**
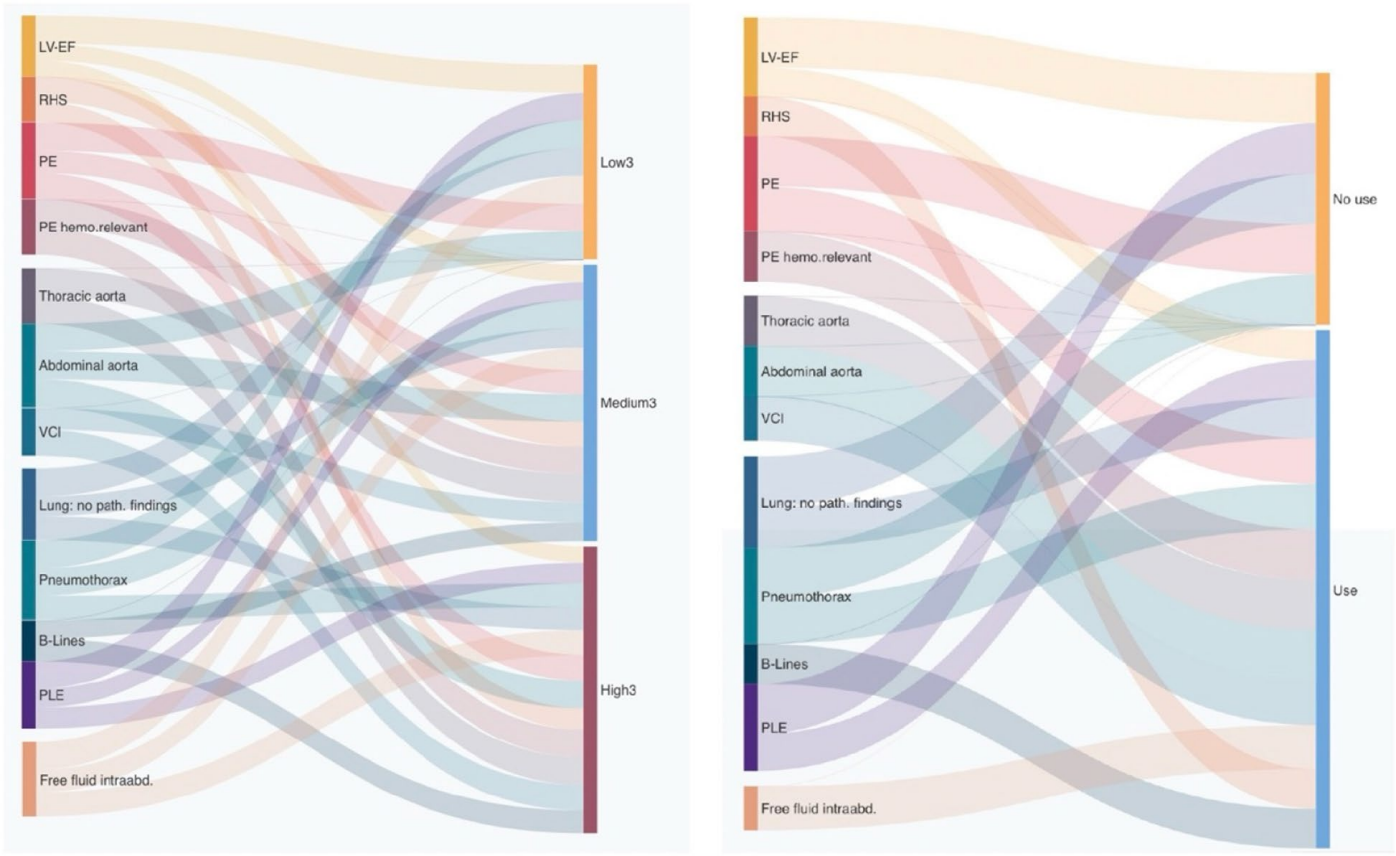
Comparison as above graphically shown as Alluvial diagrams. The connecting line widths are sized to be proportional to the number of correct prehospital sonographic findings. Left side: EMS physicians divided into three levels of competence Low3—Medium3—High3. Right side: EMS physicians divided into two levels: no use and use POCUS during in-hospital work

Table 4 shows the results of comparing the number of correct prehospital POCUS findings and the level of competence of performing EMS physicians (three competence levels: Low3—Medium3—High3 and two competence levels: *no use*—*use*). Figure 3 shows these relations graphically as an Alluvial diagram.

**Table 4:**
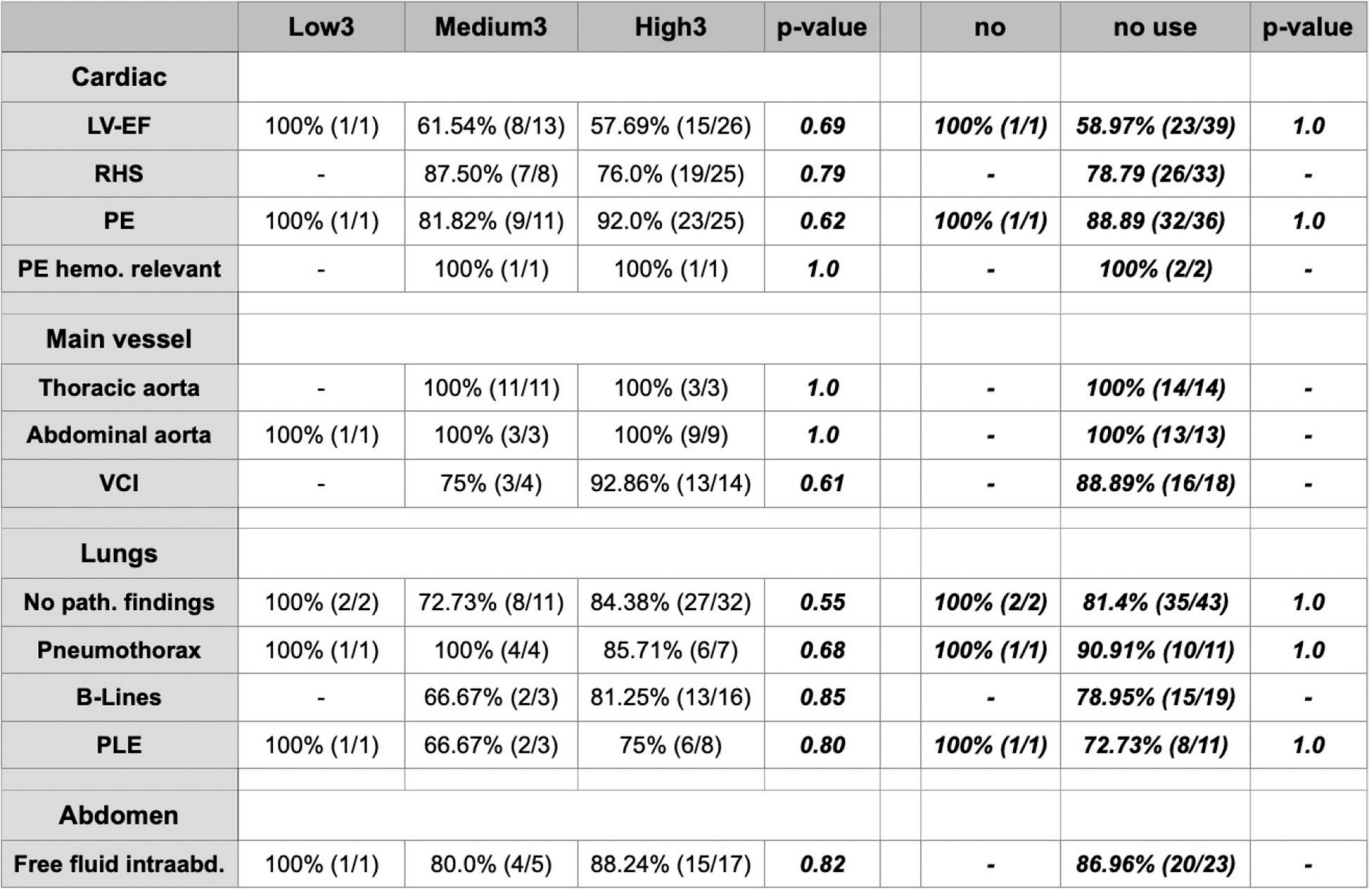
Comparison of correct prehospital POCUS findings and level of competence of performing EMS. Physicians and p-value. Three levels of competence Low3 - Medium3 - High3 (left side), and two levels of competence: no regular POCUS use (=no use) – regular POCUS use regularly (=use) (right side). - means no examinations in this subgroup, so comparison was not possible due to no comparable data

In none of the different categorizations we found any significant differences in comparison, the same result was observed when divided into two categories Low2—High2.

## Discussion

In recent years, mobile ultrasound has increasingly found its way into emergency medicine as a useful and important tool for detecting serious problems bed-side respectively on scene. The work presented here examines POCUS use in prehospital care in Germany and the need for EMS physician’s education to perform adequately.

Our data shows a wide range of indications for POCUS examinations in various leading symptoms relevant to emergency medicine. Already established in trauma patients [13], ultrasound also appears to be able to provide findings for prehospital care in many internal medical conditions too, especially by examining the heart and lungs.

Participants used mobile hand-held ultrasound devices focused on answering certain questions on scene. All examinations took less than 5 min. This time span was also demonstrated in other studies, so time on scene was not significantly extended in operations in which ultrasound examinations were performed prehospital [14]. This alleviates concerns about delays in patient care when using prehospital emergency POCUS. Regarding all this, our results are comparable to those from recent years [1, 3, 5, 15–22].

A research group from Switzerland investigated the distraction caused by ultrasound examinations of critically ill patients. They found a significant decrease in oxygen saturation went unnoticed in 75% of cases when ultrasound was used caused by focusing on POCUS examination during patient’s care. This result should be taken into account when considering whether ultrasound provides an advantage in treatment [23]. Despite the fact that on scene time is not significantly affected, ultrasound examination can distract the emergency physician`s focus, especially the inexperienced, from upcoming serious patient’s problems and immediate treatment.

Looking at the course of the patient, it was found that in addition to early diagnosis and thus more targeted therapy, the length of hospital stay of patients who have undergone prehospital sonography is shorter than that of patients who have not had an ultrasound examination which demonstrates a clear advantage of prehospital ultrasound [23].

From the author’s point of view, these data underline the important role POCUS has found in becoming an integral part of prehospital care, so it should be used universally.

We also addressed the question of what intensity of POCUS training is needed for safe use in prehospital patient care. We found a high agree of agreement when comparing our cohort’s prehospital POCUS findings with in-hospital results (79.5% in total). A high level of safety was observed, particularly in the search for critical life-threatening pathologies such as pneumothorax (correctly diagnosed in 91.7% of cases), pericardial effusion (correct in 89.2%) or free abdominal fluid after trauma (86.9%). Even in examining the left ventricular function of the heart (LV-EF) which requires a higher level of expertise than e.g. the assessment of B-lines over the lungs, a high level of agreement between the findings was observed (correct in 60%).

In the vast majority of other prehospital POCUS publications, participants have had special training in POCUS examinations before or further education during data collection (2;3;5;6). Most EMS physicians in Germany are not necessarily well-trained and specialized in using ultrasound. We compared the prehospital ultrasound findings with the results of in-hospital radiological diagnostics during emergency department care. Participating EMS physicians were divided into different levels of competence in emergency ultrasound examinations using a developed scoring system.

Unfortunately, no statistically significant difference was found between groups with different levels of expertise.

This raises the question of whether, conversely, practical experience plays no role in the correct use of emergency ultrasound. The experience gained from the application and logic say no. What we found is that even EMS physicians with a low level of expertise in ultrasound examinations seem to be able to perform POCUS correctly at the prehospital scene. It must be taken into account that the number of examinations carried out by inexperienced emergency physicians was very low compared to the number of (very) experienced physicians and only a few examination results were documented for the respective findings. Findings were correct in a high number of cases and remain in good correlation with those obtained in-hospital. Of course, safe use requires training and regular practice. However, data suggests that one does not need to be a highly trained specialist in ultrasound diagnostics to perform prehospital POCUS savely. With attended training courses (like pPOCUS—prehospital Point of Care Ultrasound) and consistent use of ultrasound (e.g. several times/month), reliable findings for patient’s care can be obtained safely. It is essential to remember that competence and training in emergency POCUS are necessary. It is not sufficient to simply roll out mobile ultrasound devices area wide. Educational training concepts like pPOCUS, focusing prehospital ultrasound use are needed.

### Limitations

When considering the results, several limitations must be taken into account. This study was a prospective observational trial. For obvious reasons no randomization or blinding was conducted. There were no guidelines for the participating EMS physicians regarding whether or which symptoms POCUS examinations should be performed prehospital. The decision was left to the respective EMS physician. The same applied to the documentation of the examinations. It cannot be ruled out that not all examinations were documented.

Follow up in-hospital data was available in only 108 cases. There was a high number of „lost cases “ due to no transport to a hospital or the inability to obtain the in-hospital findings. The four emergency ambulances in Nuremberg city complete around 23,000 missions per year (data from the rescue control center of the City of Nuremberg). Only a number of 258 POCUS examinations were documented as part of our study. Of the approximately 100 EMS physicians in the Nuremberg City ambulance service, only 32 participated in the study. Regarding this, a possibility of selection bias must be taken into account. Emergency POCUS was mostly performed by (very) experienced providers. There was just a small number of POCUS inexperienced EMS physicians. So we had limited data available for comparison between EMS physicians with different levels of competence.

Additionally, from a medical point of view, sonographic findings may already will change during prehospital treatment. The examination results in the ED may already be different, even though the ultrasound findings were correctly recorded prehospital. For example, the extent of heart failure may improve rapidly as a result of initial treatment, leading to an increased LV-EF over time, or no B-lines may be visualized over the lungs as treatment progresses.

The question of whether the study’s POCUS findings had an influence on prehospital treatment, therapeutical or organizational questions during prehospital care is part of an additional study, results are not discussed in this manuscript.

## Conclusions

The indications for the use of prehospital ultrasound extend beyond those for (poly)trauma or cardiopulmonary resuscitation. There is no significant time delay in patient care when performing prehospital POCUS on scene.

Education and training are needed, but data suggest that prehospital POCUS use can be learned quickly, with a high level of quality in obtaining the findings. Prehospital POCUS does not require a complete sonographic examination. The decisive factor is the possibility of focused diagnostics in order to identify life-threatening pathologies quickly or to rule out with a high degree of certainty.

Further studies are needed to assess POCUS influence on outcomes and mortality. In view of the experience from ultrasound use in anesthesiology, intensive care and in-hospital emergency care, we support the call for widespread mobile ultrasound for prehospital use.

## Data Availability

No datasets were generated or analysed during the current study.
